# Regulation of pulmonary plasma cell responses during secondary infection with influenza virus

**DOI:** 10.1084/jem.20232014

**Published:** 2024-04-25

**Authors:** Andrew J. MacLean, Joao P.P.L. Bonifacio, Sophia L. Oram, Mona O. Mohsen, Martin F. Bachmann, Tal I. Arnon

**Affiliations:** 1 University of Oxford, Kennedy Institute of Rheumatology, Oxford, UK; 2 Nuffield Department of Medicine, University of Oxford, The Jenner Institute, Oxford, UK; 3 Department of Bio Medical Research, University of Bern, Rheumatology, Immunology and Allergology, Bern, Switzerland

## Abstract

During secondary infection with influenza virus, plasma cells (PCs) develop within the lung, providing a local source of antibodies. However, the site and mechanisms that regulate this process are poorly defined. Here, we show that while circulating memory B cells entered the lung during rechallenge and were activated within inducible bronchus-associated lymphoid tissues (iBALTs), resident memory B (BRM) cells responded earlier, and their activation occurred in a different niche: directly near infected alveoli. This process required NK cells but was largely independent of CD4 and CD8 T cells. Innate stimuli induced by virus-like particles containing ssRNA triggered BRM cell differentiation in the absence of cognate antigen, suggesting a low threshold of activation. In contrast, expansion of PCs in iBALTs took longer to develop and was critically dependent on CD4 T cells. Our work demonstrates that spatially distinct mechanisms evolved to support pulmonary secondary PC responses, and it reveals a specialized function for BRM cells as guardians of the alveoli.

## Teaser

The authors show that following influenza virus rechallenge, lung resident, but not circulating memory B cells differentiate into PCs near infected alveoli. In these sites, outside of iBALTs, BRM cells’ activation is regulated by innate cells and signals.

## Introduction

Humoral memory is critical for the ability of the host to cope with secondary infections and is the basis for the development of most current vaccines (Plotkin, 2023). Memory B cells play a central role in this process by quickly differentiating into antibody-producing cells, thus contributing to the amplification and acceleration of secondary antibody responses (Inoue and Kurosaki, 2023). While the mechanisms that activate B cells within secondary lymphoid organs have been extensively studied (Batista and Harwood, 2009; Cyster, 2010; Dhenni and Phan, 2020; Heesters et al., 2016; Qi et al., 2014), less is known about the factors that regulate memory B cell activation in peripheral tissues.

In the lung, infection with influenza virus leads to the development of inducible bronchus-associated lymphoid tissues (iBALT) (Allie and Randall, 2017; Naderi et al., 2023; Silva-Cayetano and Linterman, 2020), where ongoing germinal center (GC) B cell responses (Adachi et al., 2015; Denton et al., 2019; Gregoire et al., 2022; Moyron-Quiroz et al., 2004; Oh et al., 2021; Tan et al., 2019, 2022) and clusters of plasma cells (PCs) (MacLean et al., 2022; Oh et al., 2021) persist for prolonged periods of time. During secondary infection, B cell responses expand in these sites, providing a local source of antibodies. However, while iBALT-associated responses play important roles in protection from secondary infection, these structures are typically found near branches of large airways and perivascular regions (Allie and Randall, 2017; Silva-Cayetano and Linterman, 2020), and they therefore occupy a relatively minor fraction of the entire lung. Since invading pathogens can infect the tissue in multiple locations, including areas that are distal to large airways or perivascular sites, it may take time before infection is detected in iBALT and antibodies reach the infected tissue.

Recent studies revealed that in addition to the well-described subset of circulating memory B cells that surveys the body by migrating between secondary lymphoid organs, a subset of non-recirculating resident memory B (BRM) cells develops and persists in previously infected lungs (Adachi et al., 2015; Allie et al., 2019; Allie and Randall, 2020; Arroyo-Díaz et al., 2023; Barker et al., 2021; Gregoire et al., 2022; Joo et al., 2008; MacLean et al., 2022; Mathew et al., 2022; Oh et al., 2021; Onodera et al., 2012; Tan et al., 2022; Weisel et al., 2020). Using a reporter mouse model and two-photon microscopy to track and visualize these cells, we recently found that while primary infection with influenza virus leads to the persistence of typical B cell compartments within iBALTs, BRM cells are not restricted to these niches (MacLean et al., 2022). Instead, they are scattered homogenously throughout the parenchyma in close association with alveoli. This spatial organization of BRM cells creates a network of cells that collectively cover large volumes of the tissue, poised to detect infection, and respond to it even in distant areas of the lung. During rechallenge, BRM cells that interface with sites of viral replication rapidly increase their migration speeds, accumulate within infected foci, and subsequently differentiate into PCs directly where viral replication is taking place (MacLean et al., 2022). The unique cellular and molecular composition of infected alveoli suggests that alternative mechanisms evolved to support BRM cell activation within them. However, little is known about the factors involved.

Here, we used our established reporter mouse model to trace memory B cell and PC differentiation during secondary infection with influenza virus. We show that the development of PCs from BRMs in the alveoli precedes their differentiation within iBALTs, and we demonstrate that this process is mediated by distinct cellular mechanisms that are induced locally in response to infection. Our work highlights the notion that within the lung, multiple microanatomical niches evolved to support the activation of memory B cells, providing an effective strategy to protect this large and fragile organ from secondary infections.

## Results and discussion

### Resident, but not circulating memory B cells differentiate into PCs within infected foci

**Figure S1.**
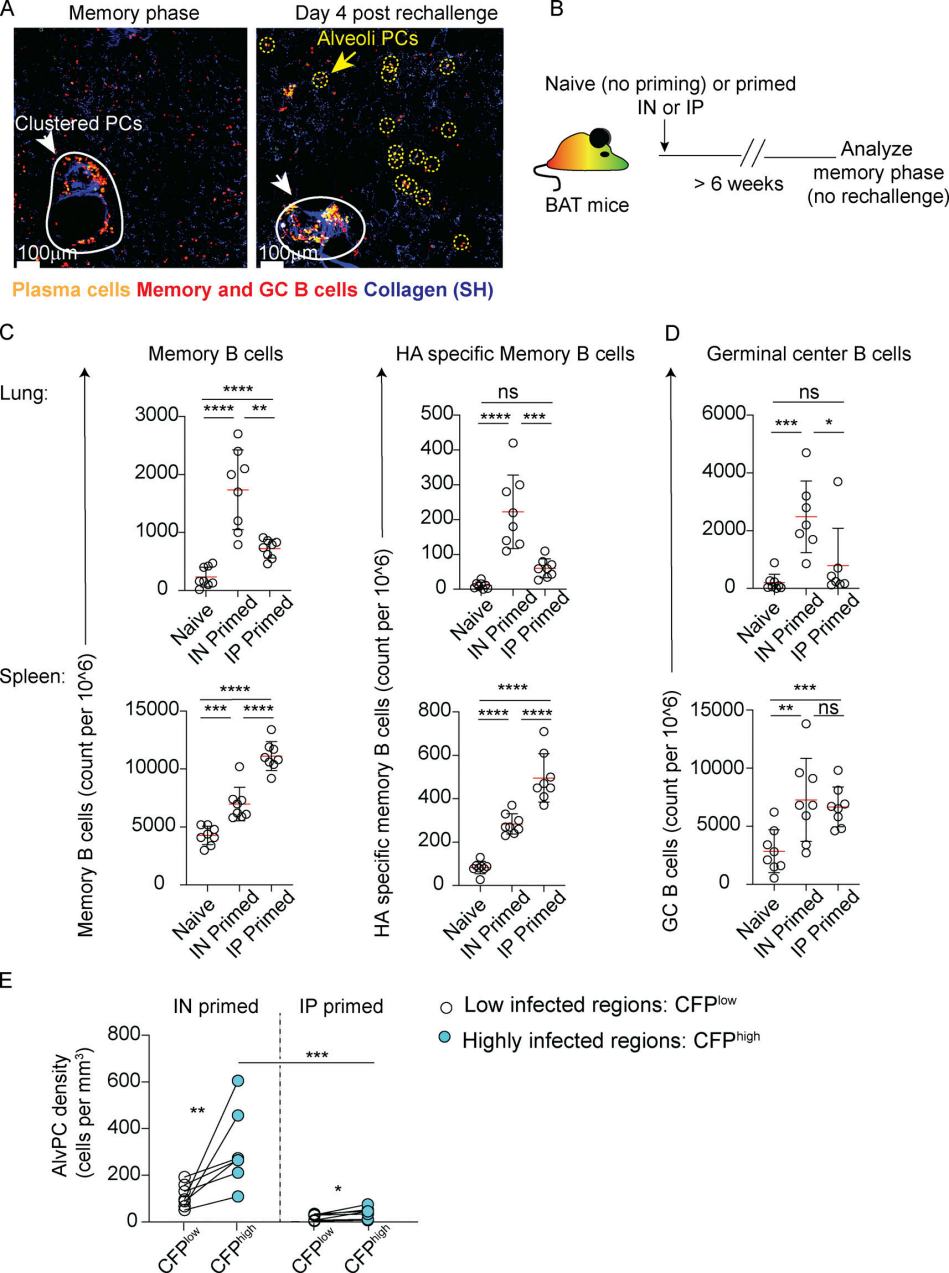
**B cell responses in the lung and spleen. (A)** Representative images from lungs of resting memory (6 weeks after infection), left, or from rechallenged BAT reporter mice, 4 days after rechallenge. Clustered PCs (white arrows and circles) and AlvPCs (yellow arrows and circles) are highlighted. The data recapitulates our recently published work (MacLean et al., 2022). **(B)** Experimental outline for data displayed in C and D. **(C)** Spleens and lungs were analyzed by flow cytometry. The figure shows quantification of total and X31 HA-specific memory B cells in naïve, IN-primed, and IP-primed mice. **(D)** GC B cell counts from lungs and spleens of mice treated as in B. **(E)** PC density in infected and uninfected areas from IN-primed or IP-primed mice at 4 days after challenge with CFP-S-flu. Analysis was performed on thick sections using static TPLSM. In C and D, data are pooled from two independent experiments, with *n* = 4 in each experiment, and each point represents one mouse. Error bars represent mean ± SD. Comparisons in C and D were made using paired Student’s *t* test. In E, each pair of points represents the average value measured in infected or uninfected regions from multiple sections (*n* = 2–5) from one mouse, representative of five independent experiments, with *n* = 1–2 per group per experiment. Comparisons between infected and uninfected regions in the same mouse (connected with lines) were made using paired Student’s *t* test. Comparisons of highly infected regions between IN and IP were made using unpaired Student’s *t* test (ns, not significant, *P < 0.05, **P < 0.01, ***P < 0.001, ****P < 0.0001).

Following primary infection with influenza virus, clusters of PCs that associate with iBALT-like structures develop and persist in the lung (MacLean et al., 2022; Oh et al., 2021). Shortly after rechallenge, new PCs develop in these sites (Allie and Randall, 2020; Chen and Laidlaw, 2022; Lee and Oh, 2022; Reusch and Angeletti, 2023), forming large aggregates that retain their confined distribution (referred in this study to as “clustered” PCs) (MacLean et al., 2022) (Fig. S1 A). We recently demonstrated that in addition to these densely packed clusters, rechallenge induces the development of another wave of PCs which distribute in the alveoli, preferentially accumulating near sites of infection. We refer to these cells as alveoli-associated PCs (AlvPCs) to distinguish them from those that occupy iBALT-like structures (MacLean et al., 2022) (Fig. S1 A). While we have previously shown that BRM cells can give rise to AlvPCs (MacLean et al., 2022), it is currently unknown whether this behavior is unique to BRM cells or if circulating memory B cells that infiltrate the lung during rechallenge contribute to this process.

**Figure 1.**
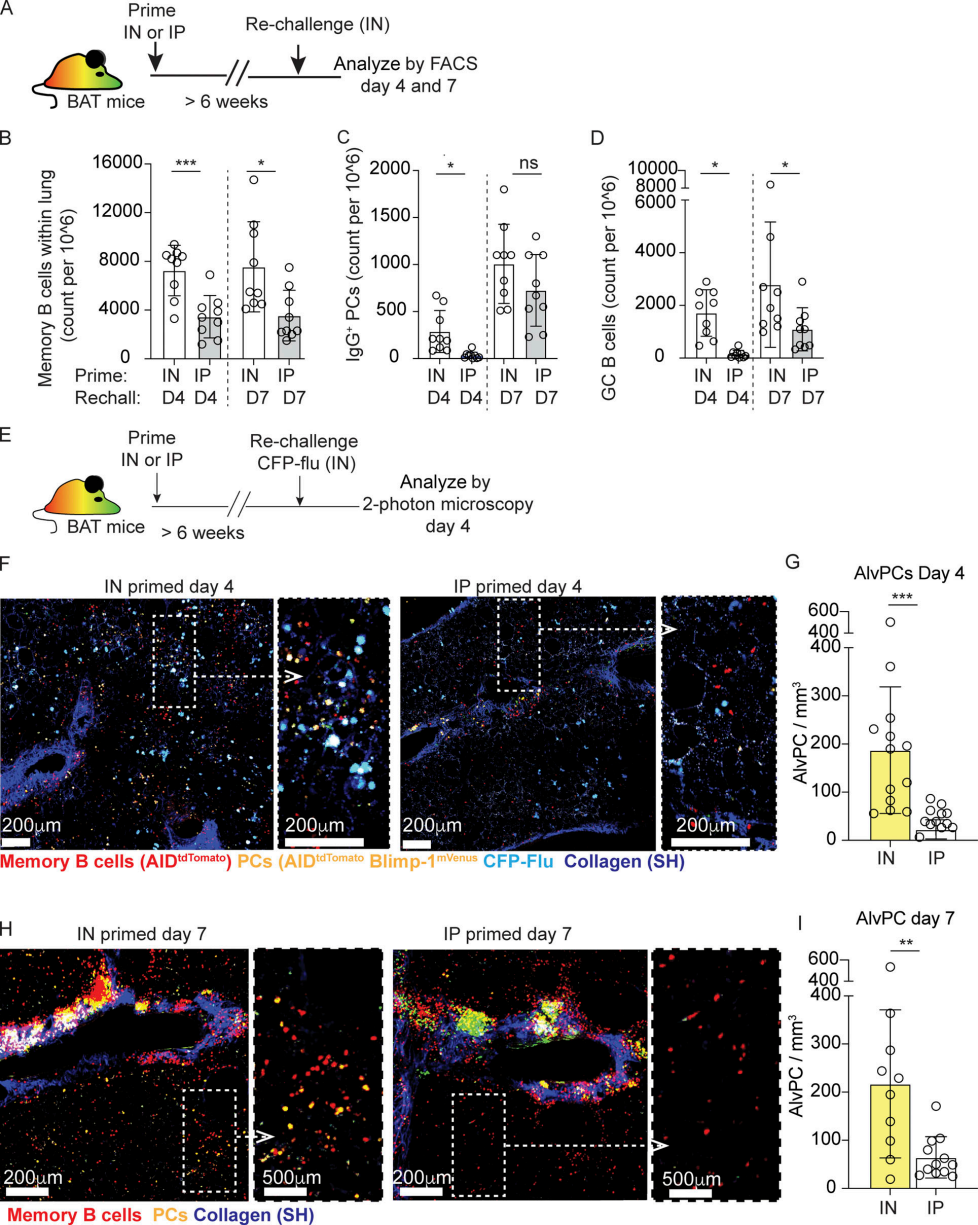
**Resident, but not circulating memory B cells differentiate into PCs within infected foci. (A–D)** (A) Experimental layout. (B) Cell counts for lung memory B cells, defined as i.v. CD45^−^ B220^+^ Blimp^mVenus−^ tdTomato^+^ GL7^−^, (C) IgG PCs, defined as i.v. CD45^−^ tdTomato^+^ B220^int/low^ Blimp^mVenus+^ intracellular IgG^+^, and (D) GC B cells, defined as i.v. CD45^−^ B220^+^ Blimp^mVenus−^ tdTomato^+^ GL7^+^. **(E–G)** Analysis of PC distribution 4 days after CFP-flu rechallenge in IN- and IP-primed hosts. **(E)** Experimental layout. **(F)** Representative images of thick lung sections collected by TPLSM. Magnification of boxed area is shown on the right. **(G)** Quantification of AlvPC density. **(H and I)** Analysis of PC distribution 7 days after rechallenge, analyzed and displayed as in F and G. Data in B–D are pooled from three independent experiments with *n* = 3–4 per experiment. Each point represents one mouse and error bars represent mean ± SD. Data in F and I are pooled from three independent experiments, total *n* = 3 per group per time point. Each point in G and I represents cells quantified from a large tiled image. Statistical comparisons throughout the figure were made using unpaired Student’s *t* test (ns, not significant, *P < 0.05, **P < 0.01, ***P < 0.001).

To address this question, we used a BLIMP1^mVenus^ AID^Cre/+^ Rosa26^tdTomato^ (BAT) reporter mouse to trace memory B cells and PCs in the lung of influenza-infected mice (MacLean et al., 2022). In these experiments, we primed the animals either intranasally (IN), to induce both local and systemic influenza-specific memory B cell responses, or intraperitoneally (IP), to primarily drive the development of circulating but not lung BRM cells (Fig. S1 B). Analysis of BAT mice at the resting memory phase (>6 weeks after infection) confirmed that, as expected (Allie et al., 2019; Bessa et al., 2008; Oh et al., 2021), IP-primed mice developed memory B cells that could readily be detected in the spleen, but not in the lung, whereas in IN-primed mice, memory B cells were induced in both sites (Fig. S1 C). A similar distribution of GC B cells was observed in spleens (Fig. S1 D). Despite low levels of lung memory B cells in IP-primed mice at rest, in response to secondary IN infection, a population of tdTomato^+^mVenus^−^GL7^−^ memory B cells was identified in the tissue within 4 days of rechallenge (Fig. 1, A and B). By day 7 after rechallenge, PCs also developed, reaching frequencies comparable with those measured in IN-primed animals at this time point (Fig. 1 C). Consistent with this pattern, lung GC B cells that were initially absent from the lungs of IP-primed mice began to expand by day 7 (Fig. 1 D). These observations show that the development of PCs in the lungs of systemically primed mice is delayed, rather than attenuated, likely reflecting the time needed to recruit circulating memory B cells to the tissue.

**Figure S2.**
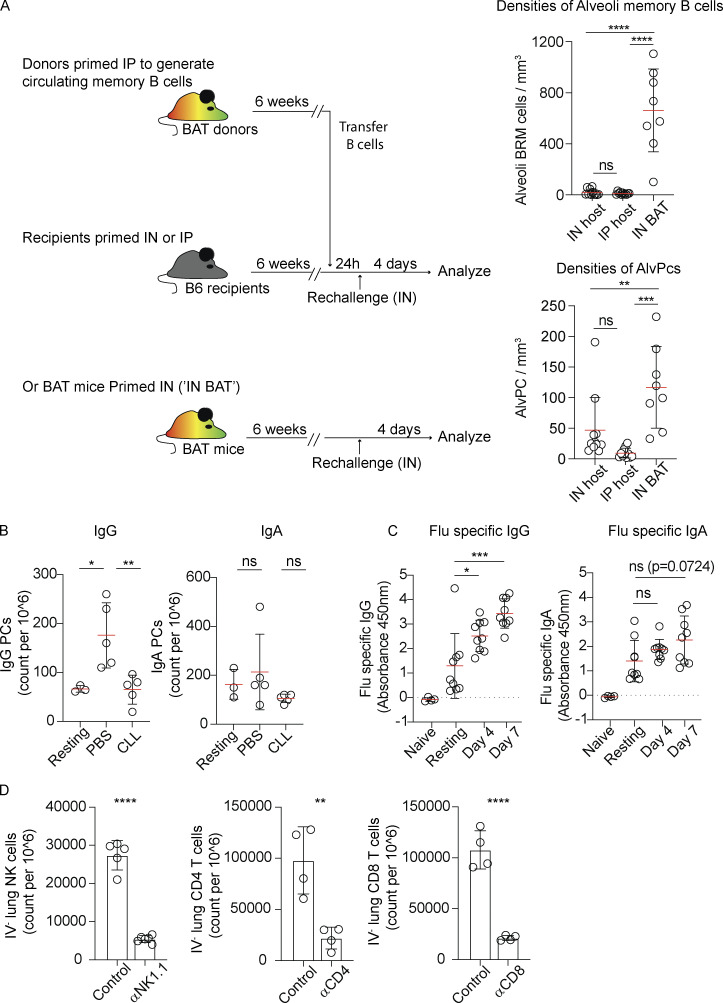
**PC development and antibody production in the lung during secondary infection with influenza virus. (A)** BAT mice were primed IP to generate circulating memory B cells. 6 weeks later, splenocytes were transferred into B6 hosts that were either IN or IP primed 6 weeks earlier. The densities of alveoli memory B cells (top) and AlvPCs (bottom) 4 days after IN rechallenge were analyzed by TPLSM. A similar analysis of BAT mice that were IN primed and IN rechallenged (“IN BAT”) is also included for reference. Data in A were collated from three independent experiments, *n* = 3 per group. Each point represents values measured from one analyzed tiled image. **(B)** Flow cytometry analysis 4 days after rechallenge showing frequencies of IgG and IgA PCs in IN-primed BAT hosts that received CLL or PBS liposomes 5 and 2 days prior to rechallenge. Figure shows one of two independent experiments performed. Each circle represents one mouse. **(C)** ELISA assay of influenza specific IgG and IgA antibodies from lungs of naïve, resting (i.e., memory phase), and rechallenged (day 4 and 7, as indicated) mice. Data in C were collected from two independent experiments. Each point represents one mouse. **(D)** Validation of depletion strategies. Frequencies of CD4, CD8, and NK cells in the lung parenchyma (i.v. CD45^−^) of rechallenged hosts, 4 days after rechallenge in the presence of absence of depleting antibodies, as indicated. NK cells were identified as B220^−^ DX5^+^ and CD3^−^, CD4 T cells as CD3^+^ CD8^−^ tdTomato^−^ mVenus^−^, and CD8 T cells as CD3^+^ CD4^−^ tdTomato^−^ mVenus^−^. Data in D relate to Fig. 2, E and F, and represent one of two and three independent experiments. Each circle represents one mouse. Statistical comparisons throughout the figure were made using unpaired Student’s *t* tests (ns, not significant, *P < 0.05, **P < 0.01, ***P < 0.001, ****P < 0.0001).

To ask if the secondary PC response in IP-primed mice localizes to infected alveoli, iBALTs, or both, we analyzed it in situ (Fig. 1 E). IP-primed mice were IN rechallenged with the CFP-S-flu, a reporter strain that allows the detection of viral foci through the induction of cyan fluorescent protein (CFP) expression in infected cells. The relative density of AlvPCs within areas displaying high (CFP^high^) versus low (CFP^low^) levels of viral infection was determined using our previously described approach (MacLean et al., 2022). Analysis of large lung sections using two-photon microscopy revealed that although circulating memory B cells in IP-primed mice could infiltrate infected alveoli within 4 days after rechallenge (Fig. 1 F), they failed to differentiate into PCs (Fig. 1 G). 3 days later, increased numbers of PCs were clearly detected in these animals, consistent with our flow cytometry data (Fig. 1 C). However, these PCs were confined to clusters (Fig. 1 H) and were rarely seen to develop in the alveoli (Fig. 1 I). This is in contrast to IN-primed animals, in which an early wave of AlvPCs developed and accumulated near infected sites already within 4 days after rechallenge (Fig. 1, F and G; and Fig. S1 E). These findings suggested that localization of BRM cells in the lung is critical for the development of AlvPCs during rechallenge. However, it was still possible that primary infection led to additional changes in the lung, which were essential for this process. To test this possibility, we transferred splenocytes from IP-primed BAT mice into *wild-type* C57BL/6 (B6) recipients that were IP or IN primed 6 weeks earlier. We found that 4 days after rechallenge, the densities of alveoli memory B cells and AlvPCs derived from the transferred cells were comparable in IP- and IN-primed recipients (Fig. S2 A), thus indicating that localization of memory B cells to the lung prior to rechallenge is required for their development into AlvPCs. Interestingly, while AlvPCs rapidly developed in IN-primed recipients, their frequencies did not increase over time, maintaining similar densities at day 4 and 7 (187.29 ± 131.48 and 217.06 ± 153.81 on day 4 and 7, respectively, P = 0.6128, comparing Fig. 1, G and I). These observations support the notion that the formation of AlvPCs primarily occurs in the early phase of the response.

Together these findings show that circulating memory B cells significantly contribute to the total mass of secondary antibody responses in the lung. However, their differentiation into PCs is delayed by at least 3 days, and their site of activation is restricted to perivascular and iBALT-like structures. Thus, BRM cells are unique in their capacity to generate an early wave of PCs that are strategically positioned in infected alveoli.

### Accumulation of BRM and AlvPC near infected sites during rechallenge is regulated by NK cells

The unusual environment in which AlvPCs develop, outside of typical clusters of lymphoid tissues, suggests that alternative mechanisms evolved to support it. We previously showed that alveolar macrophages orchestrate this process, in part by triggering a cascade of events that led to IFNγ-mediated production of CXCL9 and CXCL10, thus attracting CXCR3 expressing BRM cells. We also previously showed that the very early wave of antibodies that is generated in the lung via this pathway is dominated by preferential expansion of IgG PCs, and that this early expansion is dependent on the presence of alveolar macrophages (MacLean et al., 2022). In contrast, despite being influenza specific, IgA PCs’ development took longer to develop (Fig. S2, B and C). These observations are consistent with a model in which the initial wave of lung secondary PCs develops near infected alveoli and is dominated by IgG response, perhaps because of the highly inflammatory nature of this environment where intact virions are present, which may promote IgG switching (Chang et al., 2022; Gerth et al., 2003; Peng et al., 2002; Zabel et al., 2014; Zumaquero et al., 2019). At later times, when the development of lung PCs shifts to iBALT-like structures (Fig. 1 H), IgA responses are also induced.

**Figure 2.**
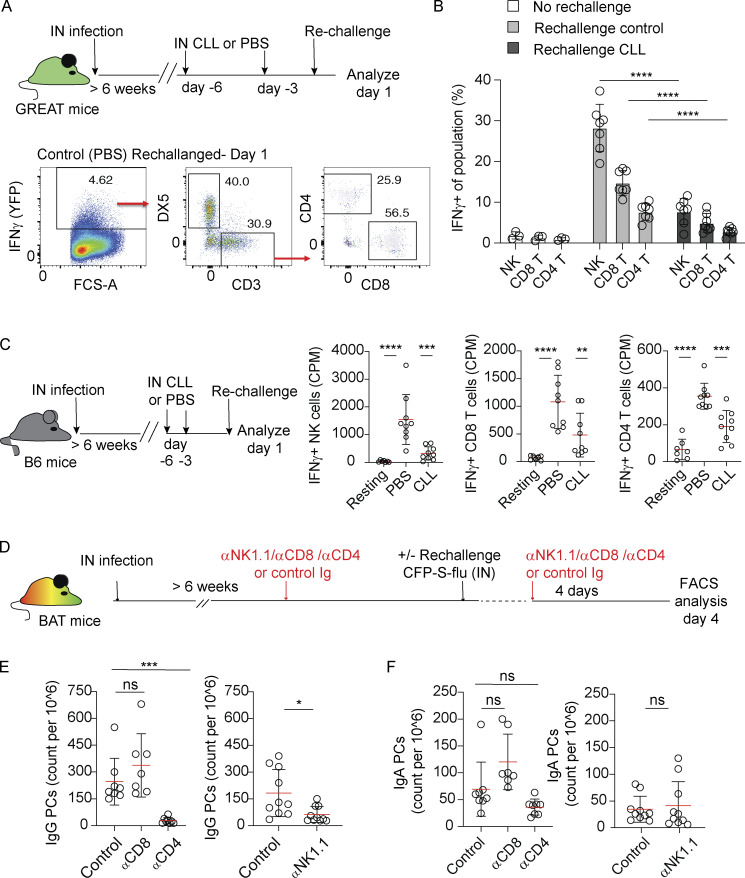
**IFNγ-producing NK cells and CD4 T cells support secondary IgG**^**+**^ **PC responses. (A)** Top, experimental layout. Bottom, representative flow cytometry plots showing frequencies of NK cells (DX5^+^ CD3^−^), CD4^+^, and CD8^+^ T cells within the pool of IFNγ^+^ cells. **(B)** IFNγ^+^ fraction of NK cells, CD4^+^, and CD8^+^ T cells in resting memory phase mice, and 1 day after rechallenge in mice treated with control or CLL liposomes, as indicated. **(C)** Left, experimental layout. Right, quantification of intracellular IFNγ expression in DX5^+^ CD3^−^ NK cells, DX5^−^ CD3^+^ CD8^+^ T cells, or DX5^−^ CD3^+^ CD4^+^ T cells analyzed by flow cytometry. **(D–F)** Analysis of PC response at day 4 after rechallenge in the absence of CD4, CD8 T, or NK cells. **(D)** Experimental layout. Lungs were analyzed by flow cytometry. The figure shows counts of (E) IgG^+^ PCs and (F) IgA^+^ PCs. Data in A, B, and C are each pooled from two independent experiments with *n* = 3–4 per experiment. Each circle represents one mouse. Data in E and F are pooled from two to three independent experiments with *n* = 3–4 per experiment. Each circle represents one mouse. Statistical comparisons throughout the figure were made using unpaired Student’s *t* test (ns, not significant, *P < 0.05, **P < 0.01, ***P < 0.001, ****P < 0.0001).

To identify the main cellular source of IFNγ that mediates AlvPC accumulation in infected regions, we assessed IFNγ expression 1 day after rechallenge in the presence or absence of alveolar macrophages. We detected IFNγ transcript levels by flow cytometry using the GREAT mouse line, in which an IRES-eYFP cassette is expressed under the control of the endogenous IFNγ promoter. Our analysis confirmed that 1 day after rechallenge, natural killer (NK) cells, CD8 T cells, and CD4 T cells were major sources of IFNγ (Fig. 2 A). Alveolar macrophage depletion reduced IFNγ production in all populations, most strikingly in NK cells, where up to two-thirds of IFNγ^+^ cells lost expression (Fig. 2 B). Similar results were obtained when an IFNγ antibody was used to directly monitor protein levels (Fig. 2 C). These observations indicate that during secondary infection, alveolar macrophages are necessary for triggering IFNγ expression in multiple cell types, suggesting that multiple cellular sources of IFNγ may be involved in the positional regulation of BRM cells and the subsequent formation of AlvPCs.

To test this possibility, we injected BAT reporter mice with depleting antibodies against NK cells, CD8, or CD4 T cells prior to rechallenge. 4 days later, at a time when PC expansion is primarily induced in infected alveoli, we assessed PC frequencies (Fig. 2 D). Depletion of each subset was confirmed by flow cytometry (Fig. S2 D). We found that the level of IgG^+^ lung PCs during rechallenge was unaffected by depletion of CD8 T cells. However, upon removal of CD4 T cells or NK cells, IgG^+^ lung PC responses were impaired (Fig. 2 E). In contrast, the numbers of IgA^+^ PCs were not reduced by these depletions (Fig. 2 F), consistent with the notion that IgG AlvPCs dominate the first wave of the recalled PC response.

**Figure 3.**
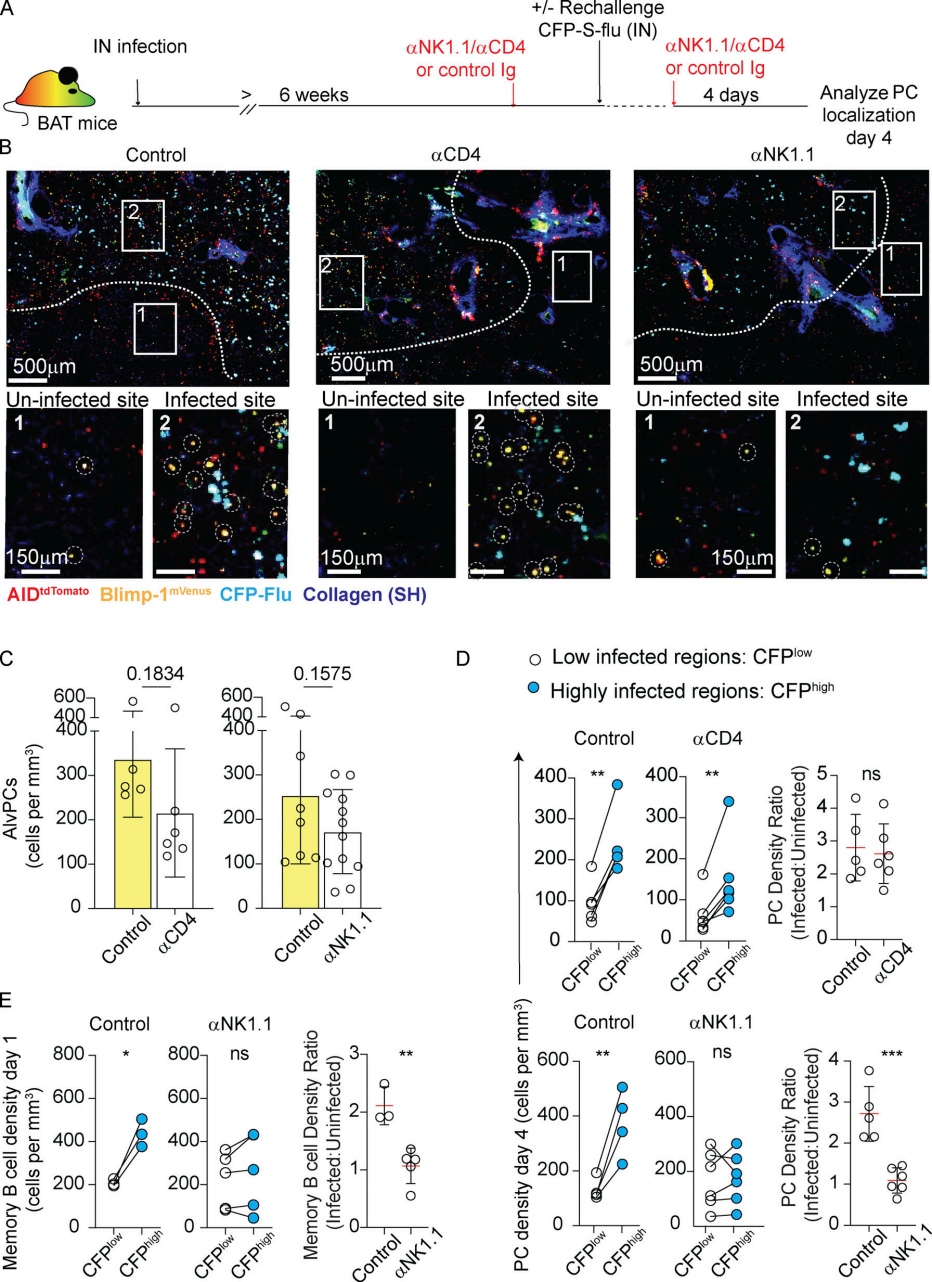
**Accumulation of BRM cells near infected sites during rechallenge is regulated by NK cells. (A–D)** Effect of NK and CD4 T cell depletion on AlvPC positioning in rechallenged lungs. **(A)** Experimental layout. **(B)** Representative images from thick sections using static TPLSM. The white line highlights an approximate border between areas that are highly versus poorly infected. Boxes focus on uninfected (box 1) and infected (box 2) areas and are displayed as magnified images in the lower panel. Dotted white circles mark AlvPCs. Each image represents one tiled of three independent experiments with *n* = 3 per group per time point. **(C)** Quantification of AlvPC density, calculated using high-volume static TPLSM imaging from images as above. Each point represents cells quantified from a large tiled image analyzed from three independent experiments. *n* = 3 per group per time point. **(D)** PC density at day 4 rechallenge, in infected and uninfected areas of control Ig-treated, anti-CD4–, or anti-NK1.1–treated mice. On the right, data displayed as the ratio of PC density in infected versus uninfected regions. **(E)** Density of BRM cells in infected and uninfected areas 24 h after rechallenge in mice treated with control Ig or anti-NK1.1 antibodies. Right, data displayed as the ratio of BRM cell density in infected versus uninfected regions. Data in D and E are each pooled from three to five independent experiments and *n* = 1–2 per condition per experiment. Each point represents multiple tiled images (*n* = 2–5) from one mouse. Data in C were compared using unpaired Student’s *t* test. Data in D and E were compared using paired Student’s *t* test (left, center plots) and unpaired Student’s *t* test (plots on the right comparing ratios) (ns, not significant, *P < 0.05, **P < 0.01, ***P < 0.001).

These findings suggested that CD4 T cells and NK cells may act together as potent IFNγ sources, potentially reflecting a non-redundant functional cooperativity between these cell types. To test this possibility, we analyzed the spatial distribution of lung PCs 4 days after rechallenge (Fig. 3 A). We found that while depletion of CD4 T cells was associated with a consistent tendency of reduced total density of AlvPCs in the tissue (Fig. 3, B and C), there was no defect in their positional regulation (i.e., accumulation near infected sites). Thus, in CD4 T cell–depleted animals, AlvPCs could be readily detected and their density in infected regions was two- and threefold higher compared with the density of these cells in uninfected areas, a ratio that was comparable with that which we measured in control rechallenged hosts (Fig. 3 D, top panel). In contrast, depletion of NK cells not only led to a decline in total density of AlvPCs in the lung (Fig. 3 C), but also resulted in a robust impaired localization to infected foci, as reflected by the lack of preferential accumulation of AlvPCs near these regions (Fig. 3 D, bottom panel).

To further challenge these results and to test the potential role of NK cells in controlling the positioning of early AlvPC precursor cells in infected regions, we examined the distribution of BRM cells at 24 h after rechallenge, at a time when BRM cells accumulate within infected foci but have not yet differentiated into PCs (MacLean et al., 2022). Our analysis confirmed that in the absence of NK cells, BRM cells failed to accumulate within highly infected regions (Fig. 3 E). We conclude that NK cells, but not CD4 T cells, are critical for BRM cell localization to sites of viral replication, and we demonstrate that when this step is defective, the capacity of preimmunized mice to develop AlvPCs is suboptimal.

### CD4 T cells are critical for PC development in iBALTs

The above results show that CD4 T cells are not required for AlvPCs accumulation near infected regions. However, depletion of CD4 T cells prior to rechallenge was associated with a robust decline in lung-localized PC numbers after rechallenging (Fig. 2 E). Previous studies demonstrate that a subset of CD4 T cells that develops and persists within iBALTs of influenza virus–infected hosts plays a central role in the maintenance of ongoing GC B cell responses (Son et al., 2021; Swarnalekha et al., 2021). Since persisting PCs reside within these sites and further increase in numbers during rechallenge, we hypothesized that CD4 T cell contribution to the total mass of PCs in the lung may be primarily mediated by their role in supporting PC differentiation within iBALTs.

**Figure 4.**
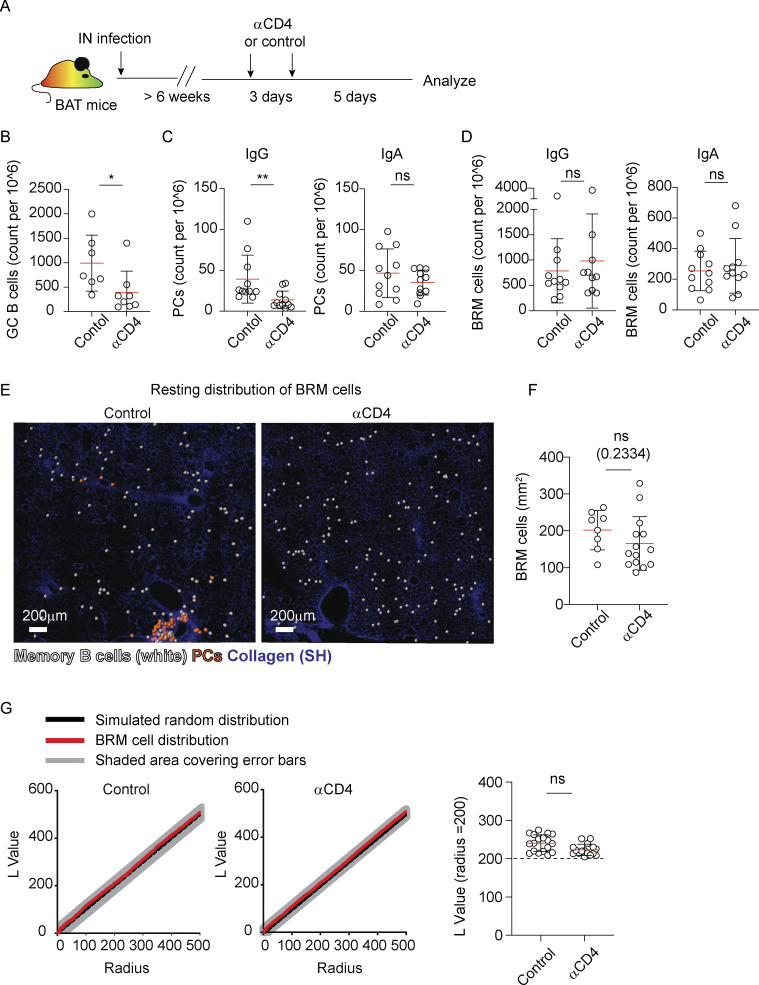
**CD4 T cells are critical for PC development in iBALTs. (A)** Experiment layout. **(B–D)** (B) Counts of GC B cells, (C) counts of IgG and IgA PCs, and (D) counts of IgG and IgA BRM cells analyzed by flow cytometry 1 week after CD4 T cell depletion in memory phase mice. **(E)** Representative images from lungs of memory phase BAT reporter mice 1 week after treatment with control or anti-CD4 antibodies, highlighting BRM distribution through the alveoli. Analysis was performed on thick sections using static TPLSM. BRM cells (white) and PCs (orange) are highlighted using spots created by Imaris. **(F)** Quantification of BRM density in lungs of mice treated as in A. **(G)** Distribution of BRM cells in control and anti-CD4 treated mice. Left, plots display clustering (Ripley’s L) values of observed data (black) versus complete spatial random simulation (simulation *n* = 1,000; red). Right, a summary of L values of observed BRM distribution at r = 200 µm. Data in B–D are representative of three independent experiments with *n* = 3–5 per experiment. Each point in B–D represents one mouse. Data in E–G are pooled from two independent experiments, total *n* = 3. Each point in F and G represents values measured from one analyzed tiled image. Statistical comparisons were made using Mann–Whitney U tests (B–D) or unpaired Student’s *t* test (F and G) (ns, not significant, *P < 0.05, **P < 0.01).

To probe this hypothesis, BAT mice were infected with influenza virus, and 6 weeks later, we depleted CD4 T cells (Fig. 4 A). Consistent with previous studies (Son et al., 2021; Swarnalekha et al., 2021), within a week after depletion, a rapid loss of GC B cell responses was observed (Fig. 4 B). PCs, which are confined to iBALT-associated clusters during the memory phase, also declined (Fig. 4 C). In contrast, the frequencies of BRM cells in the lung remained intact (Fig. 4 D). Analysis of large volumes of tiled lung sections using two-photon microscopy further demonstrated that depletion of CD4 T cells had no effect on the overall distribution of BRM cells during the memory phase as the cells remained scattered throughout the alveoli (Fig. 4 E) and their density was similar to that which we measured in control mice (Fig. 4 F). To quantitatively assess BRM cell distribution, we used the Ripley’s K function, as previously described (MacLean et al., 2022). This algorithm allows us to measure patterns of cell scattering and clustering, by comparing the normalized Ripley’s K L values calculated in experimental data sets (black line) to a simulated random distribution (red line). This analysis confirmed that the distribution of BRM cells outside of iBALTs during the memory phase was random, as we have previously shown (MacLean et al., 2022), and that this pattern was not changed by depletion of CD4 T cells (Fig. 4 G).

Taken together, the above results demonstrate that CD4 T cells play a critical role in the long-term maintenance and expansion of iBALT-associated PCs. However, their presence is not required for BRM cell survival and retention in the alveoli during the memory phase, and their contribution to the formation of AlvPCs in these sites is limited.

### Innate signals can promote AlvPC development in the absence of specific antigen

**Figure 5.**
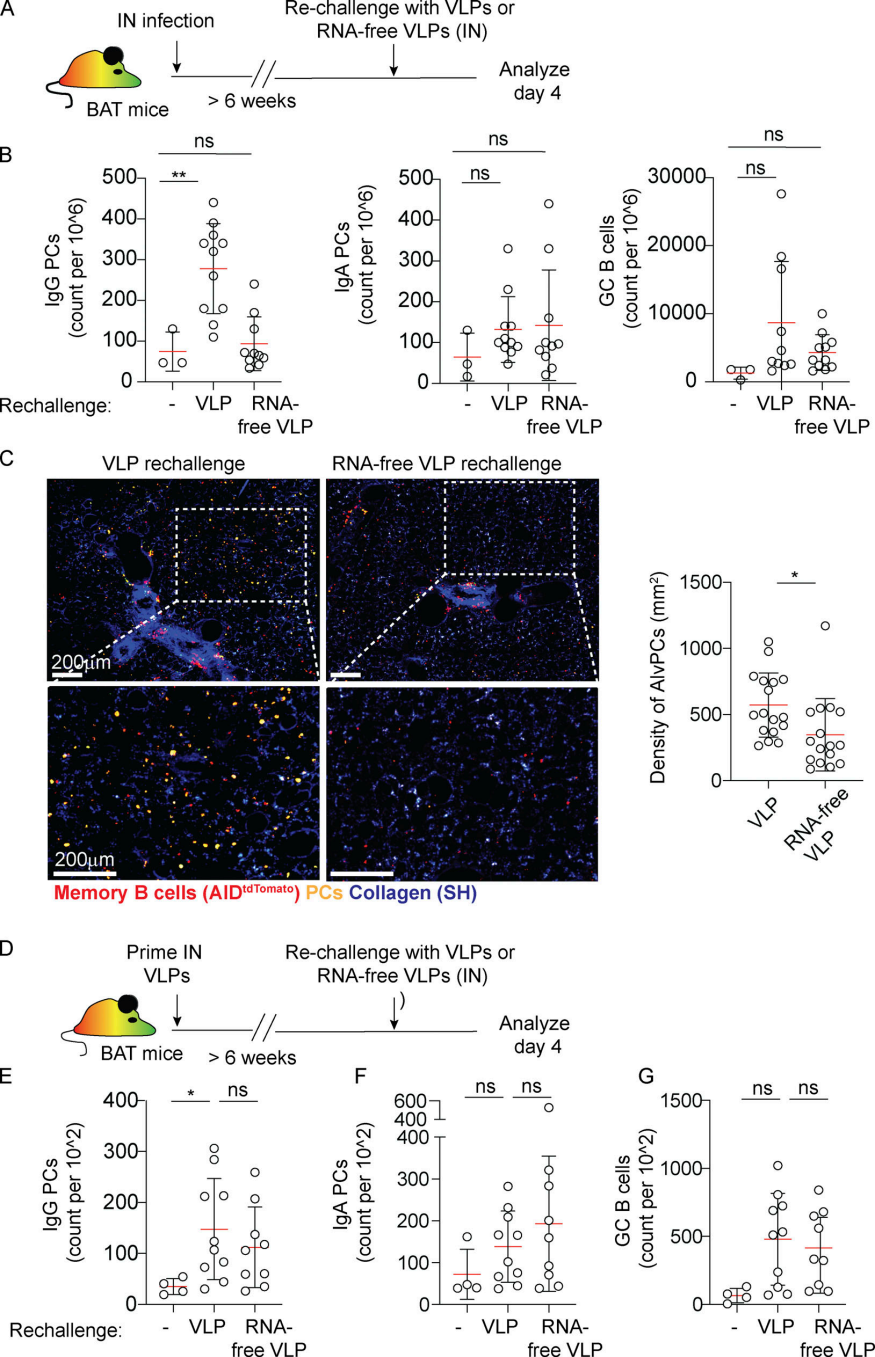
**AlvPC development from BRM can be driven by innate signals. (A–C)** PC responses in the lungs of mice rechallenged with VLPs or RNA-free VLPs. **(A)** Experiment layout. **(B)** Lungs were analyzed by flow cytometry before or 4 days after secondary challenge with VLPs or RNA-free VLPs. Shown are counts of IgG PC, IgA PC, and GC B cells. **(C)** Left, representative images from lungs of VLP or RNA-free VLP-challenged BAT reporter mice, highlighting AlvPC distribution 4 days after challenge. Right, density of AlvPC within challenged lungs. Analysis was performed on thick sections using static TPLSM. **(D)** Experiment layout for data displayed in E–G. **(E–G)** Mice were primed with Qβ-VLPs and 6 week later rechallenged with RNA-free VLP or intact VLP. Lungs were analyzed by flow cytometry before or 4 days after rechallenge. Shown are counts of IgG PC (E), IgA PC (F), and GC B cells (G). Data in B and E–G are each pooled from two independent experiments, *n* = 3–6. Each point represents one mouse (B, E–G). Data in C are pooled from three independent experiments (*n* = 3 per group); each point represents average values measured from one analyzed tiled image. Statistical comparisons throughout the figure were made using unpaired Student’s *t* tests (ns, not significant, *P < 0.05, **P < 0.01).

The observation that AlvPCs could be induced in the absence of CD4 T cells led us to ask whether this process is strictly dependent on antigen specificity, which typically requires T cell help, or whether the abundance of innate stimuli that are likely encountered in infected regions can drive this process independently of the presence of a cognate antigen. To address this question, we utilized bacteriophage-derived virus-like particles (Qβ-VLPs) (Bessa et al., 2008). These structures contain single-strand RNA (ssRNA) molecules that are loaded into the VLPs during packaging in *E. coli*, and which provide strong innate stimuli. As these VLPs share no antigenic epitopes with influenza virus, we used them to ask if AlvPC differentiation can be mediated by providing strong innate signals in the absence of a cognate antigen. BAT mice that were infected with influenza virus to induce BRM cells were rechallenged IN 6 weeks later with Qβ-VLPs (Fig. 5 A). Flow cytometry analysis revealed that within 4 days after rechallenge, the number of IgG^+^ PCs in the lung was increased (Fig. 5 B). To ask if innate stimuli regulate this effect, we rechallenged the mice with RNA-free Qβ-VLPs. Under these conditions, IgG PC expansion was severely impaired (Fig. 5 B). Two-photon analysis of large, thick lung sections further confirmed that secondary challenge with intact, but not RNA-free Qβ-VLPs, induces a clear population of sparsely distributed PCs within the alveoli (Fig. 5 C). Little or no effect on the IgA^+^ PC and GC B cell responses was observed when hosts were rechallenged with either intact or RNA-free Qβ-VLP (Fig. 5 B). These results suggest that in the absence of strong antigenic stimulation, innate cues play a dominant role in driving the activation of BRM cells within the alveoli. To test whether innate signals are similarly essential in case of secondary exposure to the same antigen, we next immunized BAT mice with Qβ-VLPs to induce VLP-specific BRM cells. 6 weeks later, we rechallenged the animals with the same particles (Fig. 5 D). Under these conditions, both intact and RNA-free Qβ-VLPs induced an increased IgG response, although a stronger effect was observed when mice were rechallenged with complete particles (Fig. 5 E). IgA PCs and GC B cells also expanded, although these responses did not reach a significant level (Fig. 5, F and G). Thus, in the presence of a cognate antigen, innate signals enhance but are not essential for reactivation of BRM cells in the lung. Taken together, our findings raise the possibility that positioning of BRM cells directly within sites of infection may expose them to high levels of innate signals, subsequently contributing to broadening the range of their response.

Our study highlights the multilayered immunity that evolved to protect the lung. The lung supports life by facilitating the oxygenation of tissues. This critical role depends on the function of the alveoli, delicate air-filled circular units that mediate gas exchange. While each individual alveolus is relatively small, collectively, the total surface area that they cover is immense. It is estimated that the lung of an adult human contains ∼300 million alveoli, covering a surface area of up to 80 m^2^ (Weibel and Gomez, 1962). iBALTs, which are primarily located near branches of large airways and perivascular sites, act as central hubs where local immune responses are induced and where pathogens are neutralized. However, pathogens that breach this barrier and reach deeper regions within the parenchyma can infect the alveoli. Given the critical role of these structures in sustaining life, minimizing damage to them is of utmost importance. We propose that BRM cells evolved a specialized function acting as guardians of the alveoli, ensuring that at the earliest time points after secondary infection, these delicate structures that oxygenate our bodies are protected.

During primary IN infection, PCs form in the lung, but these responses are relatively slow, taking ∼2 weeks to develop (Allie et al., 2019; Joo et al., 2008), and they are confined to iBALTs (MacLean et al., 2022; Oh et al., 2021). In contrast, in IP preimmunized mice, PCs were not detected in the lung after primary immunization, but their development was induced following IN rechallenge, leading to the induction of large PC clusters within 1 week. As the BRM pool in these animals is absent, we speculate that circulating memory B cells quickly infiltrated infected lungs and contributed to this process. These findings agree with recent works highlighting the capacity of circulating memory B cells to enter infected tissues and locally differentiate into PCs within them (Mao et al., 2022; Oh et al., 2019). However, while differentiation of iBALT-associated PCs did not require pre-existing BRM cells, development of AlvPCs near infected alveoli was critically dependent on this cell subset. Moreover, the formation of AlvPCs in these sites preceded the expansion of PCs in iBALTs, occurring already within 4 days after rechallenge. Why circulating memory B cells that entered the lung failed to differentiate into AlvPCs remains unknown, but it is possible that the delay in reaching infected sites prevents them from receiving necessary signals that are transiently induced during the early phase of viral replication. It will be interesting to explore this question and to further test whether circulating memory B cells that lodge to infected alveoli during IN booster vaccines, seed the tissue, and establish residency in these sites.

The finding that BRM cells persist outside of iBALTs suggests that they can be maintained independently of these structures. Supporting this possibility is our observation that depletion of CD4 T cells, which are necessary for the maintenance of iBALTs during the memory phase, had no effect on the number, density, and positional distribution of BRM cells. Furthermore, recent studies have shown that BRM cells develop and persist in the lung of *Streptococcus pneumococcus* infected mice and humans, an infectious model that does not lead to the development of iBALTs (Barker et al., 2021). Thus, while we do not argue that BRM cells are excluded from iBALTs, we conclude that these compartments are not the only niche of BRM cell residency. It is important to highlight that because BRM cells are sparsely scattered throughout the parenchyma, the detection of their collective distribution requires robust analytical approaches. It is likely that in previous studies where assessment of alveoli-associated BRM cells was missing, the analysis failed to detect the cells due to limited depth and narrow fields of view of the sections imaged (Gregoire et al., 2022; Tan et al., 2022). Our approach of using two-photon microscopy combined with a fate mapping mouse model and quantitative methods to systematically capture and assess changes in BRM cells and PC localization in the lung facilitated this analysis. The development of additional tools that will enhance the detection of these rare cells and allow following their differentiation in situ will be of great value.

It is currently unclear why IgG, but not IgA PCs, rapidly declined following depletion of CD4 T cells during the memory phase. Whether these differences reflect the existence of a specialized niche that supports IgA PC durability in the lung, as was suggested in humans (Madissoon et al., 2023), or whether they relate to differences in the mechanisms that drive their differentiation remains unknown. In line with the latter possibility, recent work demonstrates that in Peyer’s patches (PPs), IgA signaling confers a competitive advantage during GC B cell selection, allowing low-affinity B cells to persist and contribute to the PC compartment (Raso et al., 2023). While the mechanistic basis for the selective superiority of IgA signaling in PPs compared with other lymphoid organs is not clear, environmental factors may contribute. It will be interesting to test whether IgA signaling provides a similar advantage in the lung and to further explore the phenotypic and functional characteristics of BRM and PC cells in distinct niches in this organ.

In previous works, we showed that induction of AlvPCs near infected alveoli depends on a CXCR3-mediated migratory step that leads to rapid accumulation of BRM cells within foci of infection. We further showed that alveolar macrophages are necessary for subsequent induction of IFNγ, which stimulates expression of the CXCR3 ligands, Cxcl9 and Cxcl10. Here, we provide evidence supporting a critical role for NK cells as a major source of IFNγ that acts downstream to alveolar macrophages to regulate BRM cell positioning. By monitoring the magnitude and the location of the response, we show that depletion of NK cells impaired the ability of BRM cells to accumulate in infected regions and to form AlvPCs within them. NK cells evolved to quickly find and eliminate virally infected targets (Björkström et al., 2022; Forberg et al., 2014; Frank and Paust, 2020). We propose that this ability of NK cells to directly interact and kill infected cells places them in an ideal location to alter the chemotactic landscape around newly infected regions and attract BRM cells to them. Our findings point to IFNγ as being a major factor in this process, but it is possible that additional chemokines that are induced or expressed by NK cells contribute to this process. Future works will aim to address this question.

VLPs are potent immunogens with even few particles leading to a robust activation of B cells, independently of costimulation from T cells (Bachmann and Jennings, 2010; Brooks et al., 2023). We previously showed that VLPs containing ssRNA can induce BRM cell motility even in the absence of cognate antigen, suggesting capacity to regulate chemotactic signals (MacLean et al., 2022). Here, we find that this form of innate stimulation can also trigger the differentiation program of PCs near infected sites in the absence of cognate antigen. We speculate that the localization of BRM cells directly within infected regions exposes them to relatively high concentrations of intact virions, potentially allowing them to respond to a broad range of threats. In line with this possibility, we found that activation of BRM cells can be mediated via alternative mechanisms: either via cognate interactions or through innate signals delivered by viral particles, like VLPs. These findings align with previous works indicating that BRM cells contain a sizable population that displays a sub-threshold affinity to the immunizing antigen, and which upon activation tends to favor their differentiation into IgG^+^ PCs over entry to the GC (Gregoire et al., 2022). Thus, we hypothesize that the benefit of inducing a highly localized response when facing a low affinity or even an irrelevant antigen may override the potential energy loss associated with producing unnecessary antibodies.

Our study reveals the unique capacity of BRM cells to become activated throughout parenchymal regions in the lung, where during infection local inflammatory mediators and a diverse cellular network combine to promote tissue immunity. These findings further highlight the notion that even within one organ, multiple niches evolved to ensure that the entire tissue is protected. It remains to be determined whether similar mechanisms evolved to guard other organs from diseases.

## Materials and methods

### Mice

Male and female mice aged 8–16 weeks were used for all experiments. C57BL/6 (B6, CD45.2^+^) or B6 Ly5.2 (CD45.1^+^) mice were purchased from Charles River. AID-Cre (Robbiani et al., 2008) (007770; B6.129P2Aicdatm1[cre]Mnz/J) and Rosa26-stop-tdTomato (007914; B6.CgGt[ROSA]26Sortm14[CAG-tdTomato]Hze/J) and GREAT (Reinhardt et al., 2009) (IFNγ reporter with endogenous polyA transcript, 017580; C.129S4[B6]-Ifngtm3.1Lky/J) were from Jackson Laboratories. Prdm1^mVenus^ (BLIMP1^mVenus^) mice were from RIKEN (accession number CDB0460T) as described previously (Ohinata et al., 2008). BAT mice experiments were performed using marrow chimeras, in which lethally irradiated C57BL/6 mice were reconstituted with bone marrow from BLIMP1^mVenus+^ AID^Cre/+^ Rosa26stop-tdTomato^+^ animals. We used this approach to allow generation of a large cohort of mice with the correct phenotype, needed for the study, thus reducing the overall number of mice generated for the work. It is important to note that we have previously validated this model and have excluded the possibility that the use of irradiated hosts affected BRM cell development, distribution, motility, or alveolar macrophage–dependent differentiation into AlvPCs because these parameters were similar in irradiated and non-irradiated mice (MacLean et al., 2022). To generate chimeras, 8–12-week-old C57BL/6 mice were lethally irradiated (11Gy) in two dosages separated by 4 h, followed by injection of >5 × 10^6^ Blimp1^mVenus^ AID^Cre/+^ Rosa26stop-tdTomato^+^ bone marrow cells per mouse. Animals were bred and maintained under specific pathogen–free conditions at the Kennedy Institute of Rheumatology, University of Oxford. Experiments were in accordance with the UK Scientific Procedures Act (1986) under a Project License authorized by the UK Home Office.

### Influenza infections

Mice were primed IN with 2 × 10^4^ PFU X31 or IP with 1 × 10^7^ PFU of X31 influenza virus. For rechallenge experiments, 1 × 10^6^ PFU X31 was administered IN. Fluorescent CFP-S-Flu was produced as previously described (MacLean et al., 2022).

### Cell depletion

For depletion of NK cells during rechallenge, we injected anti-NK1.1 (Clone PK136, BE0036; BioXCell) or mouse IgG2a (Clone MOPC-173, cat. 400289) as control. Memory phase mice received an i.v. administration of 500 μg 1 day prior to rechallenge and an additional i.v. injected dose of 100 μg 1 h after rechallenge.

For depletion of CD4 and CD8 T cells during rechallenge, cells were depleted using anti-CD4 antibodies (Clone GK1.5, BE0003-1; BioXCell), anti-CD8β antibodies (Clone 53-5.8, BE0223; BioXCell), or control rat IgGb (Clone RTK4530, cat. 400667; Biolegend). Antibodies were injected into memory phase mice 3 days prior to rechallenge via two routes simultaneously: i.v. with 500 μg and IN with 100 μg. Another dosage of 100 μg was administrated i.v. 1 h after rechallenge.

For depletion of CD4 in resting state, mice were treated with anti-CD4 antibodies (Clone GK1.5, BE0003-1; BioXCell) or control rat IgG (Clone RTK4530, cat. 400667; Biolegend). Antibodies were administered twice: in the first dosage, animals were given 500 μg of antibody i.v. and 100 μg IN 8 days prior to analysis, and in the second dosage animals were given 100 μg IN 5 days prior to analysis.

Alveolar macrophages were depleted using IN administration of clodronated liposomes (CLL). 45 μl dosage of Clodrosome (Liposoma BV) was administered IN twice 6 days prior to rechallenge and twice 3 days before rechallenge. The selective depletion of alveolar macrophages using this approach was based on previous works (Leemans et al., 2001) and was optimized in our own hands as described in our recent publication (MacLean et al., 2022). It should be noted that we cannot exclude the possibility that small amounts of CLL that may not been sufficient to kill interstitial phagocytes have reached the parenchyma, potentially compromising neutrophil functionality (Culemann et al., 2023).

### VLPs

Qβ-VLPs were produced in *E. coli* and purified by chromatography, as previously described (Bachmann and Jennings, 2010). For digestion of VLP RNA, Qβ-VLPs were buffer-exchanged to 20 mM HEPES, pH 7. VLPs were then concentrated to 2 mg/ml and incubated at 37°C for 3 h with a final concentration of 1 mg/ml RNase A (Cat# R4875; Merck). Degraded RNA and RNase were removed by diafiltration against 20 mM HEPES followed by PBS. After diafiltration, RNA removal was confirmed by running a native agarose gel with nucleic acid stain.

Priming with VLPs was performed by IN, administering two doses of 50 µg VLPs in 30 µl PBS 2 weeks apart. To rechallenge, mice received IN instillation of a single dose containing 100 µg VLP or 100 µg RNA-free VLP in 30 µl PBS.

### Flow cytometry

Lung digestion was performed as previously described (MacLean et al., 2022). Briefly, mice were in vivo labeled with i.v. anti-CD45 antibodies 4 min before euthanization. Lungs were perfused with PBS and roughly dissected before incubation in collagenase D (1 mg/ml, Cat# 11088858001; Roche) and DNaseI (10 μg/ml, Cat# DN25; Merck) at 37°C for 45 min. Tissue was homogenized through a 70-μm filter and stained in FACS buffer (2% FBS, 0.1% sodium azide, 1 mM EDTA in PBS). Fragment crystallizable regions of an antibody block was performed for 15 min and staining for 30 min at 4°C. Cytofix/Cytoperm Staining Buffer Kit (BD Biosciences) was used for intracellular staining of antibody isotypes. Data acquisition was performed using a BD Fortessa X20 (BD Biosciences) and analyzed using FlowJo v10.8 (Tree Star, Inc.).

For intracellular antibody staining of IFNγ, cells were digested as above, resuspended in RPMI with 10 μg/ml brefeldin A, and incubated at 37°C for 3 h. Cells were subsequently processed as detailed above for antibody isotype staining.

### Influenza hemagglutinin (HA) production and biotinylation

The production of X31 HA, its biotinylation, and the confirmation of its specificity were recently reported (MacLean et al., 2022).

### Imaging static lung sections

All quantitative assessments of BRM cell or PC distribution, including calculations of clustering L values and cell densities in the alveoli or in uninfected and infected sites, were done by analyzing large tiles of static lung sections using two-photon excitation laser scanning microscopy (TPLSM), as previously described (MacLean et al., 2022). Briefly, after euthanization with pentobarbital, mouse lungs were inflated with 1 ml of 30–35°C low-melt agarose (1% in PBS) into the lungs. Individual lobes were dissected and stored in PBS before sectioning at 450 mm on a vibratome (Campden Instruments Ltd.). Sections were mounted on plastic coverslips using Vetbond tissue glue (3M), placed on ice in PBS, and immediately imaged. Sections were imaged on a Zeiss LSM880 microscope, using a primary excitation wavelength of 930 nm, with an Apochromat 20 × 1.0 DIC VIS-IR D = 0/0 (UV) objective lens.

### Image analysis

Two-photon microscope images were collected in Zen Black and analyzed using Imaris software (Bitplane, v9.3.1).

For calculation of Ripley’s K statistic, we used an approach that we recently developed and published (MacLean et al., 2022). Images were collected from large tiles of static lung sections (Z = 80–150 μm) using TPLSM. The Imaris Spots function was used to define BRM cells, PCs, and CFP^+^ infected cells based on fluorescence intensity in tdTomato, mVenus, and eCFP channels. Spots were manually verified before being exported and combined with image metadata to supply an R script with cell coordinates and image boundaries that allowed the generation of XY point patterns. The spatstat package in R was used to calculate Ripley’s K and L values using Kest and Lest functions. In plots displaying L values, each circle represents the mean L value calculated for one large tiled image (± SD).

Quantification of cell localization within infection foci was based on our recently developed and published method (MacLean et al., 2022). Images were collected from large tiles of static lung sections (Z = 80–150 μm) using TPLSM, as above. Points based on the fluorescent signal from CFP-S-Flu were generated, and these coordinates and image parameters were exported for use in the spatstat package in R, where the quadrat-count function was used to assess the density of infected cells across the tiled image, and areas with infected cells at a density of >225 cells/mm^3^ were classified as CFP^+^ regions. BRM and PC densities within these regions were quantified using a similar approach. In all cases when quantifying cell positioning in large tiles, at least two tiled images per animal were analyzed and means were plotted.

### ELISA

ELISA assays were performed as previously described (MacLean et al., 2022). Briefly, influenza-specific IgG and IgA were detected by coating Nunc-immmuno MaxiSorp 96-well plates (10394751; Thermo Fisher Scientific) with formalin-inactivated X31 influenza overnight at 4°C. Plates were blocked with 1% BSA in PBS+0.05% Tween20 at room temperature (RT) for 1 h and then serially diluted samples were incubated at RT for 2 h. Detection was performed by adding biotinylated Goat Anti-mouse IgG (405303; Biolegend) or biotinylated Rat Anti-mouse IgA, clone RMA-1 (407004; Biolegend) followed by Streptavidin-HRP incubation. (Jackson ImmunoResearch). Ultra-TMB-ELISA Substrate (34028; Sigma-Aldrich) was used for development, and optical densities were quantified using a SpectroSTAR Nano microplate reader at 450 nm (BMG Labtech).

### Adoptive transfer experiments

BAT mice were IP immunized. 6 weeks later, splenocytes were pooled and transferred in a 1:1 donor:recipient ratio into a cohort of B6 mice that were IN infected or IP immunized with X31 influenza 6 weeks earlier.

### Statistical analysis

Statistical parameters including number of mice and number of replicates are described in figure legends. Error bars represent the mean ± SD throughout. Where normal distribution could be determined, comparisons between groups were made using unpaired Student’s *t* tests. When comparing infected and uninfected areas from the same mice, we used paired Student’s *t* tests (ns, not significant, *P < 0.05, **P < 0.01, ***P < 0.001, ****P < 0.0001).

### Online supplemental material

Fig. S1 shows the overall distribution of lung PCs before and after rechallenge, the frequencies of memory and GC B cells in the spleen and lung of naïve, IN-, or IP-primed mice, and the distribution of AlvPCs in rechallenged animals that were primed IN or IP. Fig. S2 shows the capacity of adoptively transferred memory B cells to form AlvPCs in rechallenged animals that were primed IN or IP, the formation of IgG and IgA PCs and influenza-specific antibodies in rechallenged mice, and the depletion efficiency of NK cells, CD4 T cells, and CD8 T cells in the lung of rechallenged mice.

## Data Availability

Data are available in the article itself and its supplementary materials and are also available upon request from the corresponding author.
